# Supplementary material for: Predictability and stability testing to assess clinical decision instrument performance for children after blunt torso trauma
Source: PLOS Digit Health. 2022 Aug 8;1(8):e0000076. doi: 10.1371/journal.pdig.0000076 (PMC9931266; doi:10.1371/journal.pdig.0000076)
Supplement: S1 Table — (DOCX) [file pdig.0000076.s001.docx]

## S1 Table. Iterative Random Forest permutation importance scores.

| **Rule** | **Permutation importance** |
| --- | --- |
| Glasgow Coma Scale score = 15 | 0.094 |
| Thoracic Trauma | 0.09 |
| Abdominal Trauma or Seatbelt Sign | 0.09 |
| Hypotension | 0.089 |
| Emesis/retching | 0.083 |
| Costal tenderness | 0.073 |
| No Abdominal Tenderness | 0.073 |
| Mechanism of Injury = Motor Vehicle Collision | 0.071 |
| Decreased breath sounds | 0.064 |
| Age < 2 | 0.062 |
| Mechanism of Injury = Pedestrian/bicyclist struck by moving vehicle | 0.047 |
| Mechanism of Injury = Fall from an elevation | 0.045 |
| Abdominal Distention or Abdominal Pain | 0.035 |
| Mechanism of Injury = Object struck abdomen | 0.034 |
| Mechanism of Injury = Bike collision/fall | 0.032 |
| Mechanism of Injury = Motorcycle/ATV/Scooter collision | 0.018 |
