# Supplementary material for: Predictability and stability testing to assess clinical decision instrument performance for children after blunt torso trauma
Source: PLOS Digit Health. 2022 Aug 8;1(8):e0000076. doi: 10.1371/journal.pdig.0000076 (PMC9931266; doi:10.1371/journal.pdig.0000076)
Supplement: S2 Table — (DOCX) [file pdig.0000076.s002.docx]

## S2 Table. RuleFit

| **Rule** | **Coefficient** |
| --- | --- |
| No Abdominal Tenderness | -0.009 |
| Abdominal Trauma or Seatbelt Sign | 0.032 |
| Glasgow Coma Scale score = 15 | -0.034 |
| Mechanism of Injury = Motor Vehicle Collision | 0.001 |
