# Supplementary material for: Predictability and stability testing to assess clinical decision instrument performance for children after blunt torso trauma
Source: PLOS Digit Health. 2022 Aug 8;1(8):e0000076. doi: 10.1371/journal.pdig.0000076 (PMC9931266; doi:10.1371/journal.pdig.0000076)
Supplement: S3 Table — (DOCX) [file pdig.0000076.s003.docx]

## S3 Table. Predictor variables with 1:1 match between the two study datasets, PECARN and PedSRC.

|  | **Predictor variable** | **PECARN variable** | **PedSRC variable** |
| --- | --- | --- | --- |
|  |  |  |  |
| History | **Race** | *'Race'* | *'Race'* |
|  | **Vomiting*** | *'VomitWretch'* | *'Emesis post injury'* |
| Vitals | **Heart rate** | *'InitHeartRate'* | *'Initial ED HR'* |
|  | **Blood pressure** | *'InitSysBPRange'* | *'Initial ED systolic BP'* |
|  | **Hypotension** | *'Hypotension'* | *'Hypotension'* |
| Exam | **Thoracic wall trauma*** | *'ThoracicTrauma'* | *'Evidence of thoracic trauma (choice=None)'* |
|  | **Thoracic wall tenderness** | *'ThoracicTender'* | *'Lower chest wall/costal margin tenderness to palpation (choice=None)'* |
|  | **Decreased breath sounds*** | *'DecrBreathSound'* | *'Evidence of thoracic trauma (choice=Decreased breath sounds)'* |
|  | **Abdominal wall trauma*** | *'AbdTrauma'* | *'Evidence of abdominal wall trauma'* |
|  | **Seat belt sign*** | *'SeatBeltSign'* | *'Seatbelt sign'* |
|  | **Abdominal distention** | *'AbdDistention'* | *'Abdominal distension'* |
|  | **Costal margin tenderness** | *'CostalTender'* | *'Lower chest wall/costal margin tenderness to palpation'* |

Predictor variables that match exactly. * Included in PECARN clinical decision instrument
