# Supplementary material for: Predictability and stability testing to assess clinical decision instrument performance for children after blunt torso trauma
Source: PLOS Digit Health. 2022 Aug 8;1(8):e0000076. doi: 10.1371/journal.pdig.0000076 (PMC9931266; doi:10.1371/journal.pdig.0000076)
Supplement: S4 Table — (DOCX) [file pdig.0000076.s004.docx]

**S4 Table.** Predictor variables that were mapped between the two study datasets, PECARN and PedSRC.

|  | **Feature** | **PECARN** | **Values (PECARN)** |  | **Values (PedSRC)** | **PedSRC** | **Notes** |
| --- | --- | --- | --- | --- | --- | --- | --- |
| History | **Mechanism of injury** | *'RecodedMOI'* | Mechanism of injury (choice=Assault/struck)  Mechanism of injury (choice=Other blunt mechanism) | ↔ | Object struck abdomen | *'Mechanism of injury'* |  |
|  |  |  | Mechanism of injury (choice=MVC) | ↔ | Motor vehicle collision |  |  |
|  |  |  | Mechanism of injury (choice=Motorcycle/dirt bike crash)  Mechanism of injury (choice=ATV injury)  Mechanism of injury (choice=Golf cart injury) | ↔ | Motorcycle/ATV/Scooter collision |  |  |
|  |  |  | Mechanism of injury (choice=Bike crash) | ↔ | Bike collision/fall |  |  |
|  |  |  | Mechanism of injury (choice=Bike struck by auto)  Mechanism of injury (choice=Pedestrian struck by auto) | ↔ | Pedestrian/bicyclist struck by moving vehicle |  |  |
|  |  |  | Mechanism of injury (choice=Fall > 10 ft. height) | ↔ | Fall from an elevation |  |  |
|  |  |  | Unknown | ↔ | All other mechanisms of injury |  |  |
|  | **Age** | *'ageinyrs'* | 0-2 (years)  2-17 (years) | ↔  ↔ | 0-23 (months)  2-17 (years) | *'Age in years'*  *'Age in months'* | Converted ages in months to years with decimal points |
|  | **Abdominal pain*** | *'AbdomenPain'* | Yes  No  Other  Unknown | ↔  ↔  ↔  ↔ | Yes  No  Other  Unknown | *'Complain abdominal pain'* |  |
|  |  |  | Unable to assess | ↔ | Non-verbal  Incoherent  Intubated |  |  |
| Vitals | **GCS*** | *'GCSScore'* | 3-15 | ↔ | 3-15 | *'Initial GCS'* | Missing PECARN 'GCSScore' values replaced with 'AggregateGCS' |
| Exam | **Abdominal tenderness*** | *'AbdTenderDegree'* | Mild  Moderate | ↔  ↔ | Mild  Moderate | *'Abdominal tenderness to palpation'* |  |
|  |  |  | Severe  Unable to assess | ↔  ↔ | Severe  Limited exam (intubation/sedation) |  |  |
|  |  |  | No | ↔ | None |  |  |
