# Supplementary material for: Predictability and stability testing to assess clinical decision instrument performance for children after blunt torso trauma
Source: PLOS Digit Health. 2022 Aug 8;1(8):e0000076. doi: 10.1371/journal.pdig.0000076 (PMC9931266; doi:10.1371/journal.pdig.0000076)
Supplement: S5 Table — (DOCX) [file pdig.0000076.s005.docx]

**S5 Table.** Predictor variables that were adjudicated or left out between the two study datasets, PECARN and PedSRC.

|  | **Feature** | **PECARN** | **Values (PECARN)** |  | **Values (PedSRC)** | **PedSRC** | **Notes** |
| --- | --- | --- | --- | --- | --- | --- | --- |
| History | **Sex** | *‘Sex’* | Sex | ↔ | ✘ | Absent from data set | No adequate proxy found, so omitted from analysis |
|  | **Distracting injury** | *'Distracting*  *Pain'* | Yes | ↔ | Presence of any of following: skull fracture, facial fracture, clavicle fracture, rib fracture, pelvic fracture, femur fracture, dislocation, crush, or burn injury | Absent from dataset | We defined distracting pain in the PedSRC dataset as the presence of any of the injuries listed on the left |
|  |  |  | No  Unknown | ↔  ↔ | Absence of all of above  Absence of all of above, some missing values |  |  |
|  | **Femur fracture** | Absent from data set | ✘ | ↔ | Yes  No  Unknown | *'Femur fracture'* | No adequate proxy found, so omitted from analysis |
