# Supplementary material for: Predictability and stability testing to assess clinical decision instrument performance for children after blunt torso trauma
Source: PLOS Digit Health. 2022 Aug 8;1(8):e0000076. doi: 10.1371/journal.pdig.0000076 (PMC9931266; doi:10.1371/journal.pdig.0000076)
Supplement: S6 Table — (DOCX) [file pdig.0000076.s006.docx]

## S6 Table. Children with intra-abdominal injury requiring acute intervention predicted very low risk by the original PECARN clinical decision instrument on the PedSRC external validation dataset.

| **Age, Years (Race/Ethnicity)** | **Mechanism** | **Additional Clinical Findings** | **Intra-abdominal Injury** | **Acute Intervention** |
| --- | --- | --- | --- | --- |
| 1  (White, non-Hispanic) | Rollover MVC | Traumatic brain injury with skull fracture; femur fracture | Grade 1 liver laceration; Grade 3 splenic laceration | Exploratory laparotomy |
| 6  (Black, African American) | Fell > 10 feet | Extremity fracture | Grade 5 liver laceration | Angio-embolization |

*MVC: motor vehicle collision*
