# Supplementary material for: Predictability and stability testing to assess clinical decision instrument performance for children after blunt torso trauma
Source: PLOS Digit Health. 2022 Aug 8;1(8):e0000076. doi: 10.1371/journal.pdig.0000076 (PMC9931266; doi:10.1371/journal.pdig.0000076)
Supplement: Attachment — Submitted filename: 20220601_PLOSDigital_RevisionLetter.docx [file pdig.0000076.s013.docx]

**RE: Response to Reviewers (PDIG-D-22-00077)**

June 1, 2022

Dr. Nicole Yee-Key Li-Jessen

Academic Editor

PLOS Digital Health

Dear Dr. Li-Jessen,

We respectfully submit our revised manuscript, *Predictability and Stability Testing to Assess Clinical Decision Instrument Performance for Children After Blunt Torso Trauma,* for consideration.

We appreciate the reviewer’s comments and thoughtful feedback on using the PCS data-science framework to re-evaluate and externally validate a previously developed clinical decision instrument to identify children with intra-abdominal injuries after blunt torso trauma. We used two independent prospectively collected databases. The PCS data science framework can effectively assess and vet clinical decision instruments after development but before costly external prospective validation.

The reviewers have some questions about the proposed framework and analysis. We have addressed these concerns in this point-by-point response to reviewers and updated the manuscript.

We hope that you will find these findings of sufficient relevance and importance to merit publication.

Sincerely,

| Aaron E Kornblith MD  aaron.kornblith@ucsf.edu  Phone: (415) 476-3346  Fax: (415) 476-5360 | Chandan Singh, Ph.D  chandan_singh@berkeley.edu  Phone: (571) 315-5748  Fax: (510) 643-7846 | Bin Yu, Ph.D  binyu@berkeley.edu  Phone: (510) 642-2021  Fax: (510) 642-7892 |
| --- | --- | --- |

# Journal Requirements

i. *Please amend your detailed Financial Disclosure statement. This is published with the article. It must therefore be completed in full sentences and contain the exact wording you wish to be published. State what role the funders took in the study. If the funders had no role in your study, please state: “The funders had no role in study design, data collection and analysis, decision to publish, or preparation of the manuscript.”*

This work was supported in part by NSF TRIPODS Grant 1740855, DMS-1613002, 1953191, 2015341, IIS 1741340, ONR grant N00014-17-1-2176. Moreover, this work is supported in part by the Center for Science of Information (CSoI), an NSF Science and Technology Center, under grant agreement CCF-0939370. This project was supported in part by the National Center for Advancing Translational Sciences, National Institutes of Health, through UCSF-CTSI Grant Number UL1 TR001872. The funders had no role in study design, data collection and analysis, decision to publish, or preparation of the manuscript.

ii. *Please update your Competing Interests statement. If you have no competing interests to declare, please state: “The authors have declared that no competing interests exist.”* ([link](https://journals.plos.org/digitalhealth/s/competing-interests)).

The authors have declared the following competing interests exist.

Aaron Kornblith is a co-founder \ to CaptureDx.

Newton Addo is a co-founder of CaptureDx.

Atul Butte is a co-founder and consultant to Personalis and NuMedii; consultant to Samsung, Mango Tree Corporation, and in the recent past, 10x Genomics, Helix, Pathway Genomics, and Verinata (Illumina); has served on paid advisory panels or boards for Geisinger Health, Regenstrief Institute, Gerson Lehman Group, AlphaSights, Covance, Novartis, Genentech, and Merck, and Roche; is a shareholder in Personalis and NuMedii; is a minor shareholder in Apple, Facebook, Alphabet (Google), Microsoft, Amazon, Snap, 10x Genomics, Illumina, CVS, Nuna Health, Assay Depot, Vet24seven, Regeneron, Sanofi, Royalty Pharma, AstraZeneca, Moderna, Biogen, Paraxel, and Sutro, and several other non-health related companies and mutual funds; and has received honoraria and travel reimbursement for invited talks from Johnson and Johnson, Roche, Genentech, Pfizer, Merck, Lilly, Takeda, Varian, Mars, Siemens, Optum, Abbott, Celgene, AstraZeneca, AbbVie, Westat, and many academic institutions, medical or disease specific foundations and associations, and health systems. Atul Butte receives royalty payments through Stanford University, for several patents and other disclosures licensed to NuMedii and Personalis. Atul Butte’s research has been funded by NIH, Peraton (as the prime on an NIH contract), Genentech, Johnson and Johnson, FDA, Robert Wood Johnson Foundation, Leon Lowenstein Foundation, Intervalien Foundation, Priscilla Chan and Mark Zuckerberg, the Barbara and Gerson Bakar Foundation, and in the recent past, the March of Dimes, Juvenile Diabetes Research Foundation, California Governor’s Office of Planning and Research, California Institute for Regenerative Medicine, L’Oreal, and Progenity.

Bin Yu has consulted at Microsoft Research, Grail and GenenTech and is a minor shareholder at PatternComputer, Inc. She has received honoraria for invited talks at IQVIA, GenenTech and CapitalOne. She has been funded by CZBiohub with an intercampus research award grant.

iii. *Please provide separate figure files in .tif or .eps format only and remove any ensure that all files are under our size limit of 10MB.*

Thank you. We have updated our figure files to ensure formatting requirements.

iv. *Please review your reference list to ensure that it is complete and correct. If you have cited papers that have been retracted, please include the rationale for doing so in the manuscript text, or remove these references and replace them with relevant current references. Any changes to the reference list should be mentioned in the rebuttal letter that accompanies your revised manuscript. If you need to cite a retracted article, indicate the article's retracted status in the References list and also include a citation and full reference for the retraction notice.*

Thank you. We have not made any changes to our references.

# Reviewer Comments

We gratefully thank the reviewers for their time, effort, and thoughtful comments. After revising based on their comments, we feel the manuscript is now much improved.

## Reviewer #1

***1. Comparison with PECARN study*** *“Thank you for submitting this piece. As I understand it, the aim was to use a novel framework for data science to evaluate a current CDI. The reason for doing so is to evaluate the likelihood that the CDI would be successful when implemented prospectively. It would seem that the opposite scenario would be failure of a CDI based on the PCS framework would mean some additional work should be done before implementing a CDI.”*

*“This paper has many robust assessments of the PECARN and PedSRC data sets. A question that seemed to be raised was, what measures of Predictability, Computability, and Stability are or have been used in the past? It seemed as though algorithmic quality indicators were being used to evaluate a tool perhaps on different axes? For example, while specificity and sensitivity are used in Figure 1, the blunt trauma CDI is represented by a single point, which is slightly difficult to compare characteristically with the other curves.”*

Thanks for this question. The original PECARN study (Holmes et al. 2013) used measures of predictability including diagnostic test characteristics such as sensitivity and specificity, but not an entire sensitivity-specificity curve shown in Figure 1. The critical difference between the predictability in our work and the original PECARN study is that we use a sample-split, which ensures that our test characteristics are more reliable and do not overestimate a CDI’s performance due to overfitting. Thus, the reported test characteristics for the PECARN model on the PECARN dataset (Figure 1, black dot) are for reference to the original PECARN study, but should not be directly compared with the alternate models, which are developed only on the development set. We have clarified this in the text:

Updated Figure 1 caption: “Figure 1. Sensitivity-specificity curves for clinical decision instruments to evaluate children after blunt torso trauma on the PECARN (a) development dataset (b) internal validation dataset, and (c) external validation on the PedSRC. The clinical decision instruments were then ranked by predictability from best to worst (top to bottom). PECARN (black) represents the static test characteristics of the original study, which was developed using the entire dataset (rather than only the development set).”

In the Methods section, we updated it to say, “We followed the original PECARN development and internal cross-validation process to redevelop the PECARN CDI to identify children at very low risk for intra-abdominal injuries undergoing acute intervention.(6) We used a Classification and Regression Trees (CART) rule list,(36) which involves binary recursive partitioning using the Gini criterion.(37)” We added (Page 14, Line 8), “A critical difference of PCS and the original PECARN study is that we use a sample-split, which ensures that our test characteristics are more reliable and do not overestimate a CDI’s performance due to overfitting.”

The previous PECARN study does not report measures of stability (this one of the key new strengths of the PCS framework). It also does not report measures of computability. We have added the following line to the Methods section (Page 14, Line 25), “The original PECARN study does not report stability at the model-level or variable level.”

***2. Clarifying PCS Framework*** *“Similarly, it was not clear how or why the elements in the PCS framework are chosen and how best to calculate them. It might be that there is no specific approach to measuring these qualities, but if so, perhaps it could be highlighted in the introduction. The introduction introduced the PCS framework, but not necessarily how it was used to identify or improve other models.”*

Thank you for this comment. The specifics of predictability, computability, and stability are particular to a clinical problem. We will highlight this in the introduction as well as clarify the writing around how we measure predictability, computability, and stability.

Please see the updated Introduction section (Page 5, Line 1), “The specifics of how to measure predictability, computability, and stability are particular to clinical problem and judgment calls. Here, we show how PCS can be used to assess a CDI at the model-level and variable-level.”

***3. Actionable results of PCS framework*** *“Did the act of assessing the models in itself lead to further investigation of important variables, or does the appropriate use of the PCS framework lead to results which can be directly employed in the model? It does not seem so, but it was not clear what to expect from the use of PCS on this CDI.”*

Thank you for this important question. Assessing the models via PCS allows for measuring the stability of the findings, ensuring that the resulting CDI predictor variables can be trusted. For example, the finding that the same predictor variables are important across different models/data subsamples increases trust in these variables (and the PECARN CDI which uses them). If different important predictor variables had been found to be stable across models, then CDI may have to be adjusted to use those variables. We’ve updated the text to make this more clear:

We have updated the Introduction section, (Page 5, Line 3), “We show how PCS can be used to assess a CDI at the model-level and variable-level. First, multiple CDIs are developed by subsampling the original PECARN dataset. Second, each CDI is screened at the model-level based on diagnostic test characteristics (predictability) and interpretability. Third, we assess the variability of the importance of different predictor variables across high-performing CDIs (variable-level stability).”

***4. Direct language*** *“Overall, this was a very robust and well-written manuscript, but it might benefit from some more direct language in the introduction and methods which might then make the results more easy to interpret. It seemed as though the results themselves could not be used to directly determine if the CDI "passed" a PCS evaluation as it simply gave numbers which did not have any context with which to evaluate how good or poor the performance was or what was limiting that performance.”*

Thank you for this comment. We have made the Introduction section more clear by adding, (see #2), “The specifics of how to measure predictability, computability, and stability are particular to the context of a given problem and judgment calls.” There is no threshold at which a CDI “passes” a PCS evaluation, rather there is an accumulation of evidence for clinicians to make a decision on whether or not it the test characteristics and stable variables make sense within a clinical problem. We have added some text to make this clearer:

We have updated our introduction to stability within the Introduction section, (Page 4, Line 33), “Stability measures how much a CDI varies as choices made during the data science life cycle (including data cleaning and modeling), such as reasonable data alterations or different modeling techniques.(16) Stability is assessed by comparing model-level and variable-level test characteristics to one another.”

***5. General questions*** *“Just some general questions regarding results were that you mention you then developed a PCS CDI from three variables. Is this a necessary approach to using PCS framework or an additional, perhaps third aim? It was not clear that developing a new CDI was a direct result of the approach mentioned in the methods.”*

Thank you for this comment. The aim of the study was not to develop a new CDI, but instead, to evaluate the stability of the original PECARN CDI. The three-variable CDI emphasizes that the three main predictor variables in the PECARN CDI are stable. We have added text to clarify this:

In the Abstract (Page 2, Line 15), “Three predictor variables (abdominal wall trauma, Glasgow Coma Scale Score <14, and abdominal tenderness) were found to be stable. A CDI using only these three variables would achieve lower sensitivity than the original PECARN CDI with seven variables on internal PECARN validation but achieve the same performance on external PedSRC validation (sensitivity 96.8% and specificity 44%).”

In the Results (Page 9, Line 1), “The three predictor variables that were found to be stable on PCS testing identified 60/62 patients with intra-abdominal injuries undergoing acute interventions in the PedSRC dataset, corresponding to 100% of the PECARN CDI’s predictive power.”

## Reviewer #2

***6. Study overview*** *“This was a very well written and informative article evaluating the PECARN CDI for blunt abdominal trauma in children using the predictability computability stability (PCS) data science framework. The clinical problem the authors seek to address is very relevant. The methodology and the results are presented nicely and conclusions is valid.”*

Thank you for your comments and appreciation of the study.

## Reviewer #3

***7. Clarifying next steps*** *“Excellent Manuscript that will establish process for future studies. Thank you for completing this and submitting.”*

*“The discussion does have a section detailing what the impact is on the PECARN CDI, and if I am reading correctly it will now proceed to external validation, without additional changes or study? While this is discussed, I am left a little uncertain as to exactly what are the planned next steps - and I am sure that the readers will be looking for that clarity in the manuscript.”*

Thank you for this comment. PCS concluded that the original PECARN CDI should proceed to external validation. We had mentioned this idea in the Discussion section, but have We have now highlighted it in the conclusion statements.

In the Conclusion (Page 12, Line 19), we added: “We found that the predictive performance and stability of the PECARN CDI warranted further investigation, including prospective external validation.”

We adjusted the Abstract Conclusion (Page 2, Line 21), “The PCS data science framework vetted the PECARN CDI and its constituent predictor variables prior to external validation. We found that the 3 stable predictor variables represented all of the PECARN CDI’s predictive performance on independent external validation. The PCS framework offers a less resource-intensive method than prospective validation to vet CDIs before external validation. We also found that the PECARN CDI will generalize well to new populations and should be prospectively externally validated. The PCS framework offers a potential strategy to increase the chance of a successful (costly) prospective validation.”
